# Changes and interruptions during COVID-19: caregivers of people with brain health challenges—A qualitative analysis

**DOI:** 10.3389/frdem.2024.1360112

**Published:** 2024-03-20

**Authors:** Polly Kennedy, Carol Rogan, Dawn Higgins, Yao Chen, Emilia Grycuk, Iracema Leroi, Andrew Wormald, Miriam Galvin

**Affiliations:** ^1^Academic Unit of Neurology, Trinity College Dublin, Dublin, Ireland; ^2^Dementia Research Network Ireland, School of Medicine, Trinity College Dublin, Dublin, Ireland; ^3^Department of Psychology, Maynooth University, Maynooth, Ireland; ^4^Global Brain Health Institute, Trinity College Dublin, Dublin, Ireland; ^5^Lille CHU Univ. Lille, Inserm, CHU Lille, Lille Neurosciences & Cognition, UMR-S1172, Degenerative and Vascular Cognitive Disorders, Lille, France; ^6^Department of Psychiatry, School of Medicine, Trinity College Dublin, Dublin, Ireland; ^7^School of Nursing and Midwifery, Trinity College Dublin, Dublin, Ireland

**Keywords:** brain health, supports, disruption, distress, challenges, pandemic, caregivers

## Abstract

**Background:**

The social and economic challenges of the COVID-19 pandemic greatly impacted people's physical and mental health. The majority of care for individuals with brain health challenges, including dementia and mental illness, is provided by informal family caregivers. The “Coping with Loneliness, Isolation and COVID-19” (CLIC) Global Caregiver Survey 2020 received responses from over 5,000 caregivers across 50 countries of people living with enduring brain and/or physical health conditions.

**Aim:**

This study examined English-speaking caregivers of people with brain health challenges (dementia and mental health conditions) descriptions of changes and interruptions in their ability to provide care in the context of the COVID-19 pandemic.

**Materials and methods:**

Quantitative and qualitative data were collected as part of the large-scale CLIC Global Caregiver Survey. Data from over 900 English language respondents were analyzed using descriptive statistics and thematic content analysis. A multidisciplinary team of clinicians and health policy practitioners participated in team-based qualitative analyses.

**Results:**

The majority of respondents were from the United States (71% USA), female (83%) and care providers to people living with dementia (81%). Respondents reported concerns about their loved one's physical and mental health, the limited access to other caregiving sources and the limited opportunities to maintain personal wellbeing. Practical, social, psychological and emotional impacts affected their ability to offer care. There was clear evidence that the disruption to health and social care services—institutions, day care and home services impacted the ability to offer care.

**Discussion:**

The pandemic may be seen as a catastrophic “event” that negatively impacted lives and livelihoods. A number of the social determinants of health were negatively impacted for the caregivers surveyed during this prolonged period. Caring for caregivers and supportive health and social care interventions are required to maintain the wellbeing of this informal workforce. This study represents the largest, cross-country survey on the impact of the COVID-19 pandemic on caregivers of people with brain health challenges to date; serving as an important resource for support agencies and to inform policy.

## Introduction

Over the course of the COVID-19 pandemic, social and economic challenges affected people's health and wellbeing. Social determinants of health include a variety of factors such as economic stability, education, community and social context and health care availability (World Health Organization [WHO], 2022). The WHO defines brain health as “the state of brain functioning across cognitive, sensory, social-emotional, behavioral and motor domains, allowing a person to realize their full potential over the life course, irrespective of the presence or absence of disorders” (World Health Organization [WHO], 2022, para. 1). Brain health challenges are defined here as dementia, mental ill health and intellectual disability. Neurological disorders are the number one cause of “disability adjusted life years” (DALYs), with one the biggest contributors being dementia (Feigin et al., 2019). Globally, ~9.9 million people develop dementia each year (Prince et al., 2015a).

The majority of care provided for people living with brain health challenges including dementia, other neurodegenerative disorders and mental illness is provided by informal caregivers—often spouses or partners, adult children or other family members (Chiao et al., 2015; Prince et al., 2015b; Vatter et al., 2018, 2020; del-Pino-Casado et al., 2019). Informal caregivers are essential in supporting the physical, psychological and practical needs of individuals with brain health challenges. Caregiving is related to high levels of burden, psychological distress and reduced quality of life for caregivers, which were amplified during the COVID-19 global pandemic.

Prior to COVID-19, a majority of caregivers of people with brain health challenges experienced high levels of distress, burden, loneliness and social isolation (Beeson et al., 2000; Beeson, 2003; Byers et al., 2010; Burke et al., 2017; Galvin et al., 2018). The COVID-19 pandemic has significantly increased these impacts, particularly since these caregivers are often older and physically vulnerable themselves (Chen et al., 2021). The COVID-19 pandemic required caregivers to change their caregiving routines due to the loss of external supports and respite provision, the need for increased precautions, long periods of cohabiting with family members and the care recipient and/or of separation with caregiving recipient (World Health Organization [WHO], 2020).

The aim of this study was to explore how caregivers of people with brain health challenges described changes and interruptions in their ability to provide care in the context of the COVID-19 pandemic. The pandemic may be seen as a catastrophic “event” which negatively impacted on a number of the social determinants of their health.

## Materials and methods

The “Coping with Loneliness, Isolation and COVID-19” (CLIC) was an online global survey with over 20,000 respondents, including over 5,000 caregivers across 50 countries of people living with enduring brain and/or physical health conditions during the period June–November 2020 (O'Sullivan et al., 2021). The CLIC Global Caregiver survey was embedded within the CLIC survey and focused on the experience of informal caregivers of people with enduring health problem—physical and brain health conditions (Grycuk et al., 2022).

Recruitment took place via voluntary sector organizations, charities and social media. Survey participants were 18 years and older and provided informed consent. Ethics approval was received from Ulster University (RG3) in May 2020 and, where required, it was additionally ratified by ethics committees in participating countries.

**Figure 1:**
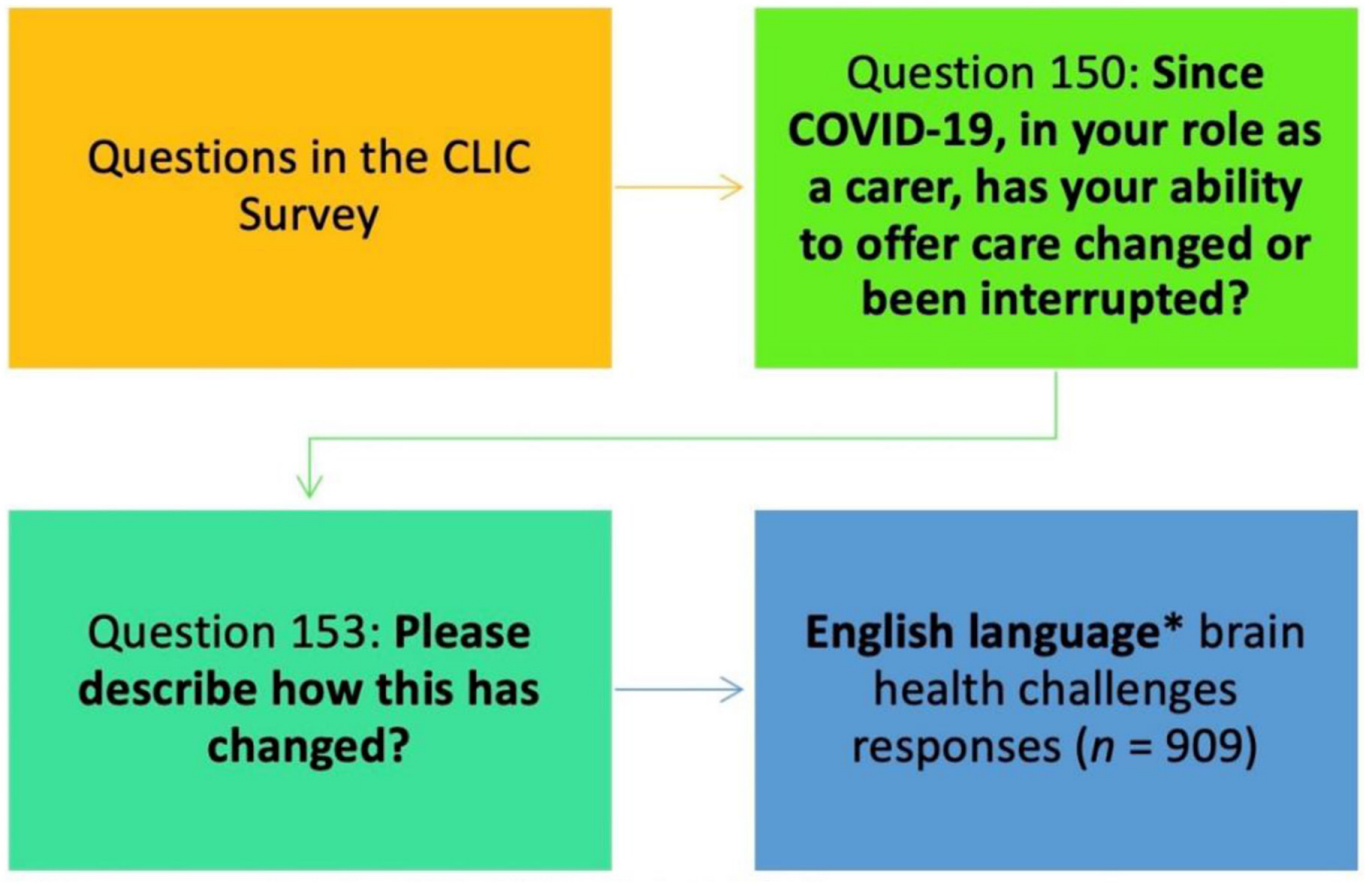
Derived sample for qualitative analysis. *English language respondents from USA, UK, New Zealand, Ireland. **Brain health challenges—Mental health and dementia.

This paper reports on data from English language survey respondents who were caregivers of people with “brain health challenges”—people living with dementia and those with mental ill health (Chen et al., 2021). Caregivers from the United States (USA), United Kingdom (UK), Ireland and New Zealand are included in this analysis. Caregivers were asked if their ability to offer care changed or was interrupted during the COVID-19 pandemic and to describe these changes and interruptions (Figure 1).

### Analysis

**Figure 2:**
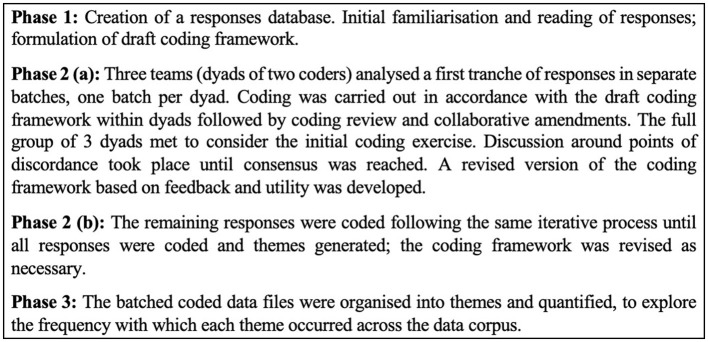
Phased qualitative analysis process.

Responses in the English language from 909 caregivers were analyzed in a number of phases (Figure 2). Text responses were qualitatively analyzed, using thematic analysis from which emerging themes become analytical categories (Braun and Clarke, 2006; Fereday and Muir-Cochrane, 2006). A team-based approach was used in coding and theme development with multidisciplinary teams of analysts from research, clinical and health service backgrounds. Each team (coding dyad) comprised of different levels of qualitative research experience and professional background adding interpretative complexity and the inclusion of multiple perspectives in researcher backgrounds provided rich opportunities to discuss coding interpretations and refinements (Berends and Johnston, 2005).

Inductive coding and theme generation was carried out using Microsoft Excel for Microsoft 365 (Microsoft Corporation, 2018). The response data were coded, patterns were identified, and themes were developed and refined. A codebook was created based on the coding of an initial batch of 150 responses −50 responses for each the three coding dyads. The codebook provided a framework for the analysis of the entire dataset, and evolved over the course of the analysis, as new codes were added, existing codes were amended, expanded and merged (Braun and Clarke, 2006). All of these events were recorded in the codebook log which was a living document over the course of the work. The development of this coding system necessitated the “recoding” and amendment of earlier work in light of details in the finalized codebook. The codebook served as a data management tool and evidence/audit trail for study credibility (Nowell et al., 2017). Flexibility and dialogue were necessary for this team-based collaborative analysis approach (Milford et al., 2017). Differences in coding and theme development were discussed and consensual agreement was reached and conceptually representative of responses in this specific context (Ulin et al., 2012). The frequency of codes and themes was then quantified across the data.

## Results

**Figure 3:**
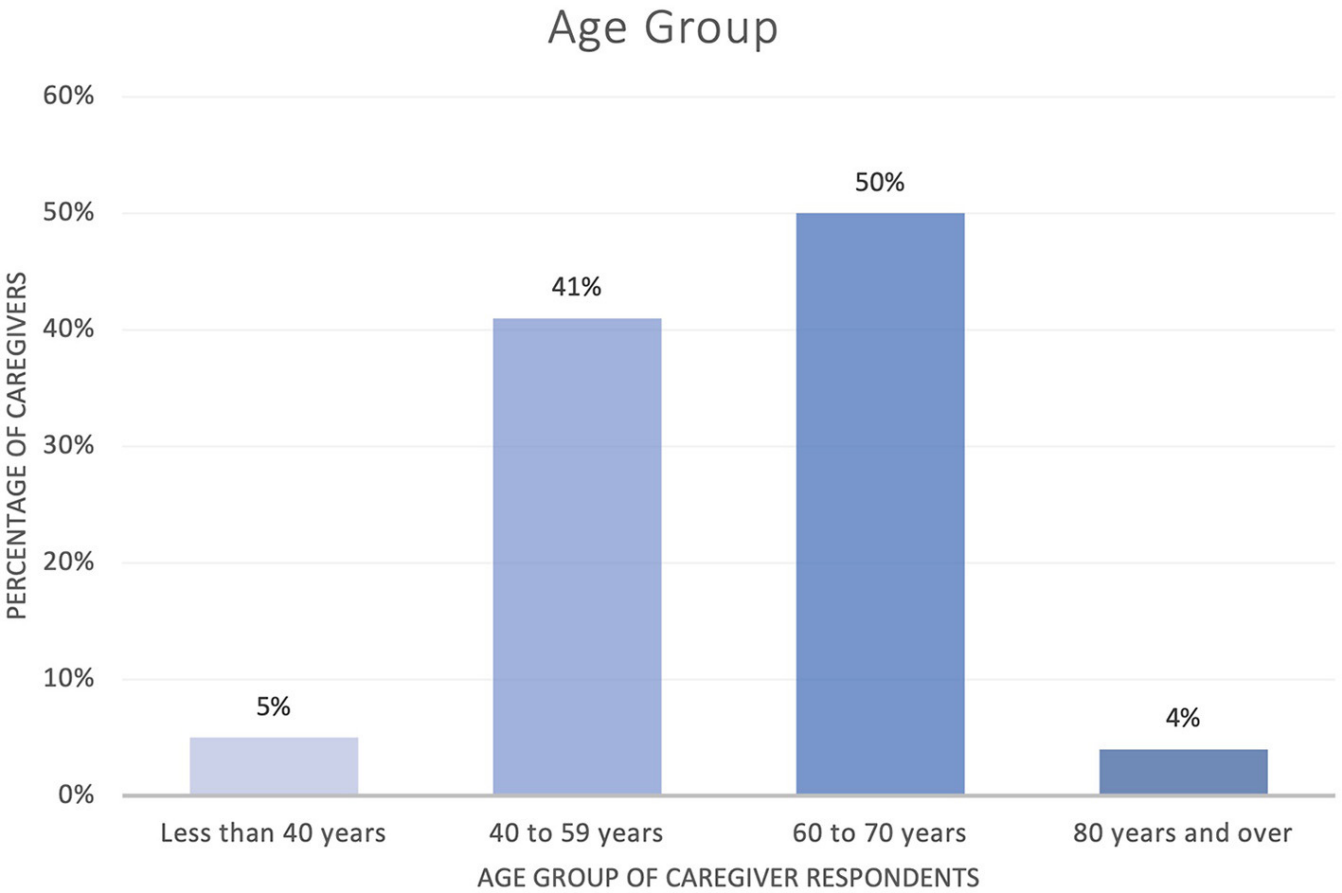
Age group of caregiver respondents (*n* = 909).

The majority of English-speaking respondents were from the USA (71% USA; 18% UK; 3% New Zealand and 8% Ireland). Eighty-one percent of respondents were caregivers of people with dementia, 15% of people with mental health difficulties and 4% with both conditions. Fifty percent of caregivers were aged between 60 and 79 years (Figure 3). Eighty-three percent of all caregivers were female. They identified predominantly as family members of the person with brain health challenges.

Approximately 90% indicated the ability to provide care had changed or been interrupted, changing “*a lot*” for almost 80% of respondents.

Over 900 respondents described how their ability to provide care changed due to COVID-19. Each response could carry more than one code. Our analysis resulted in 2,500 coded items and this paper reports on five main themes generated.

### Codes and themes

**Figure 4:**
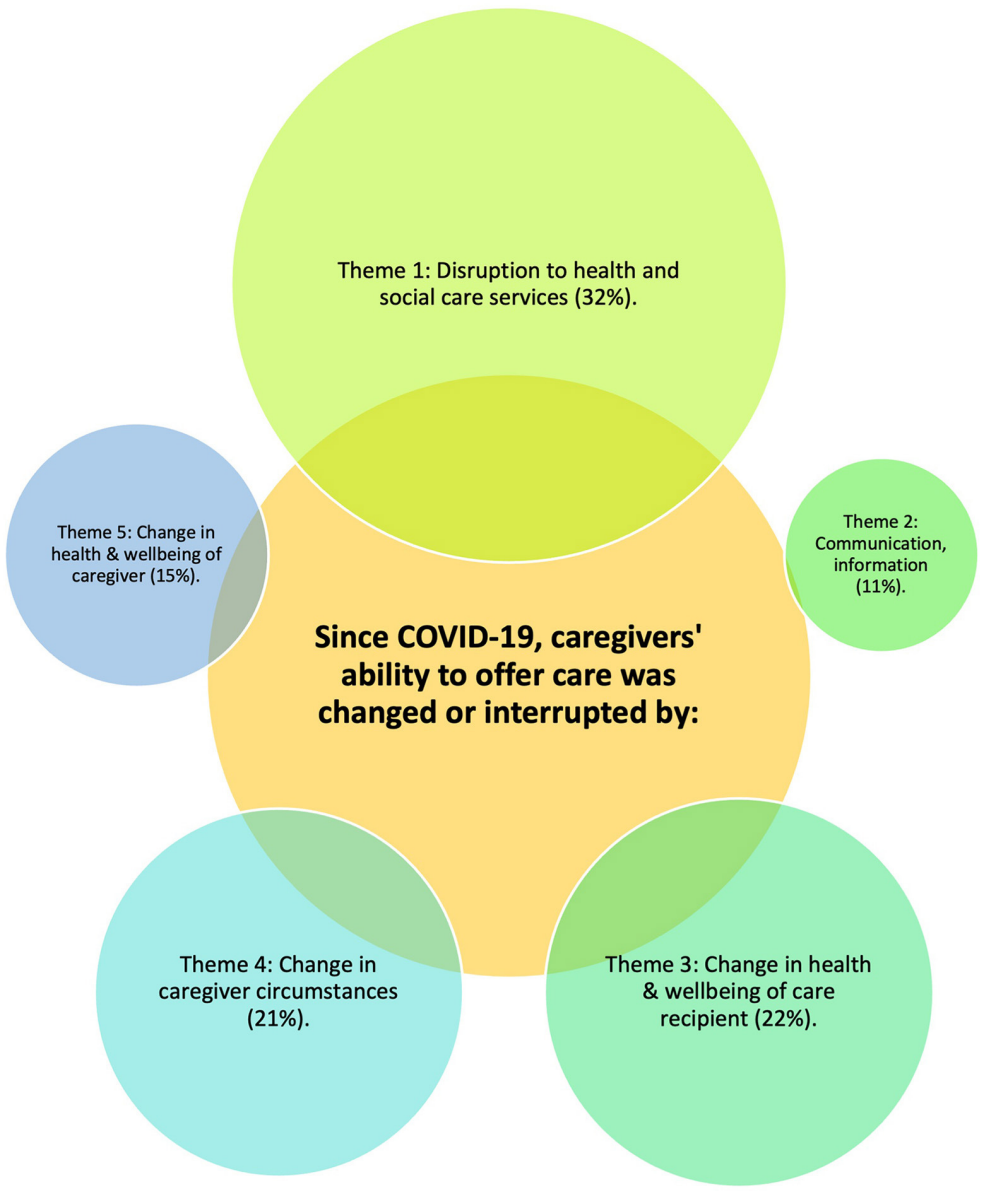
Overview of qualitative themes (*n* = 909).

For these caregivers the ability to offer care due to the COVID-19 pandemic changed or was interrupted by: (1) disruption to health and public services; (2) communication, information; (3) change in the health and wellbeing of the care recipient; (4) change in caregiver circumstances; and (5) change in the health and wellbeing of the caregiver (Figure 4).

#### Theme 1: disruption to health and public services

This theme contains codes related to multiple factors associated with disruptions to usual services especially in health and public services. Thirty-two percent of all data item codes were thematised under Theme 1. Disruptions to services “interrupted” usual routines and carers described “changed/canceled doctors” appointments, cancellation of day care services, transport restrictions, and closure of care facilities, which commonly meant less in-person contact with care partners.

Some verbatim example quotes are presented below:

“*Since lockdown her day-care ended, I stopped the (xx) carers visiting Mum and eventually she had to go into residential care in July 2020.”*

“*I find myself getting irritated with the lack of supports from health services.”*

“*Lack of support from outside services, G.P, public health nurses and carers. I feel I've been left to fend for the person who is very ill with no back up at all. It was bad pre covid, now its horrendous.”*

“*Outside carers have now stopped so caring responsibilities are solely with family now.”*

#### Theme 2: communication, information

Respondents described some positive and innovative uses of technology during the pandemic. However, access to technology and the ability to use it were obstacles for other caregivers. Technology could facilitate contact but on occasion the artificial nature of that in comparison with in-person contact was confusing for some people with impaired health and sensory difficulties which caused added distress. Wearing Personal Protective Equipment (PPE) effected communicating with loved ones, and the distance requirements made communication more difficult. Some expressed frustration at what they perceived as inadequate information provided by health services and care facilities. Communication difficulties such as these meant that some caregivers could not monitor their loved one's care situation.

Eleven percent of all codes were grouped to this theme. Some verbatim example quotes are presented below:

“*There is a great reduction in ability to observe subtle facial cues and body language through PPE and using online tools.”*

“*Interaction with facility staff is limited to short phone calls.”*

“*We stopped Skype and telephone calls which she couldn't understand & seemed to increase her confusion.”*

“*It is frustrating for him because he doesn't recognize me on the screen.”*

“*She is also hearing impaired, so electronic communication will not work.”*

“*I get no information from the home about my Mother's condition, and I am worried I will never see her alive again.”*

#### Theme 3: change in the health and wellbeing of the care recipient

During the pandemic period the health and quality of life of the care recipient deteriorated in many cases, all of which impacted or interrupted the care routine. In addition, fear, stress and loneliness during that time negatively affected the wellbeing of their loved one with brain health challenges.

Twenty-two percent of all codes were thematised here. Some verbatim example quotes are presented below:

“*Dad does not understand what is happening and he feels isolated”?*

“*…my mother does not understand the virus or the precautions we have to take. I explain it to her every day. Sometimes she gets angry, sometimes she ignores me.”*

“*… isolation and loneliness …in turn has caused a deterioration in [his] mental health.”*

“*…impact on loss of words or mental pictures has been severe.”*

“* I've noticed significant change in her appearance, her demeanour and her ability to communicate. She is more easily confused and feels like I've abandoned her.”*

“*He was overwhelmed with anxiety due to ‘End (of the) World' and terrified of my …leaving the home to work each day.”*

#### Theme 4: change in caregiver circumstances

Changes in the caregivers' own life and circumstances impacted on their ability to provide care during the pandemic. Some lost their jobs or hours of employment were reduced, there was loss of income changing the life situations of the caregivers. Those who retained their jobs, could have experienced changed working conditions and working from home. The imposed widespread restrictions on movement and lack of social engagement led to isolation for some, and dependency for others. Where young children and other family members remained at home because of school and other closures, the domestic dynamic of home care for people with brain health conditions changed.

Twenty-one percent of all codes were thematised here. Some verbatim example quotes are presented below:

“*Closure of sources of relaxation and sport. Restrictions on shopping and having to rely on others.”*

“*Our son, age 43, was sent home from group home for about four months. This made it very difficult caring for him as well as spouse who has Alzheimer's.”*

“*Having to home educate my kids …left less time for care for my mum”*.

“*Increase in living expenses: mainly food delivery cost.”*

“*Sourcing and cost of providing cover for these (home) carers was left to family.”*

#### Theme 5: change in the health and wellbeing of the caregiver

The pandemic period saw changes to the health and wellbeing of the caregiver. In particular a range of psychological and emotional effects such as stress, worry, sadness, fear and loneliness were all mentioned by respondents. These interrupted and changed his/her ability to provide care.

Fifteen percent of all codes were grouped to this theme. Some verbatim example quotes are presented below:

“*I have less tolerance and patience.”*

“*there is almost no outside world any longer.”*

“*My mental health has deteriorated and my ability to care has reduced.”*

“*We spend too much time together now and I get very tired.”*

“*It is exhausting …he cannot be left alone, and I can't bring help into the house for fear of a helper bringing the virus into our home.”*

“*a tremendous amount of time being spent in the same house. The amount of questions (the same ones) are so very repetitive. It can be very difficult to remain calm.”*

## Discussion

The lockdowns and the related levels of isolation and enforcement were aimed at limiting virus transmission, and predictably affected the daily lives of caregivers (Caulkins et al., 2021). Many countries implemented lockdowns of varying intensities and durations to control the spread of COVID-19 in their populations. There was less opportunity for social contact or to relieve stress and frustration as leisure facilities were not operating as before. There was an increased risk of social isolation, loneliness, psychological distress, and other adverse health problems for caregivers, with concurrent barriers of access to health care services and treatment of underlying conditions (United Nations [UN], 2020). Our study noted the systemic, social and personal impacts for over nine hundred caregivers living through the pandemic. Five main themes were generated through our analysis reflecting issues in the literature. Caregivers were concerned about their own physical and mental health, as reduced access to other care resources impacted care tasks and routines.

Our findings illustrate the interruptions to care provision and the impact of a range of internal and external factors on the health and wellbeing of caregivers during this period. Personal emotions coexisted with the emotional and practical challenges related to caregiving tasks (Dellafiore et al., 2022).

Some responsibilities were present more than usual; for example, the greater need to provide emotional support than before the pandemic, shopping for groceries and essentials and contacting health and social care providers. Respondents modified their caregiving approach by assuming added responsibilities, leveraging technology and managing new caregiving routines.

Employment, housing, access to education and food are among the elements considered to be social determinants of health and thus non-medical factors that influence health outcomes (Kerschbaumer et al., 2023). During COVID-19 there were altered work patterns and government supported employment policies such as flexible furlough in the US, UK and New Zealand; and a wage subsidy scheme in Ireland (World Economic Forum, 2020). Primary, second and third level educational institutions were either completely closed for significant periods or were intermittently open followed by further shutdowns. The countries represented in our study pivoted to online education, and remote working became an option for those whose work activities could be reconfigured (OECD, 2021). There was increased likelihood of families and groups of people being present in the home together, with stresses, and shortages of space and privacy (Dellafiore et al., 2022). Restriction and lack of opportunities compromised social engagement for all. Umberson and Karas Montez (2010) remind us of the links between social relationships and both short- and long-term health outcomes which continue across the life course toward cumulative advantage or disadvantage in health. Gaps in health and social care systems across countries were exposed (Rosenthal and Waitzberg, 2023).

The lack of direct contact between staff in long term care facilities and families reduced the likelihood of getting individualized information and increased the risk of formal errors (Abrams and Szefler, 2020). Our findings show some dissatisfaction with communication from health providers alongside fears associated with not able to monitor health and wellbeing of their loved ones. Challenges relating to the appropriateness of generic communication technology with people with impaired brain health were described the pandemic intensified the need for physical, psychological and social wellbeing (Hachinski et al., 2021). The importance of social interaction became clear through its absence, as personal social networks and opportunities for external engagement were limited.

The possibilities to improve health care services through telemedicine and the benefits of technology in communication and treatment through teleconsultations have been discussed (Haleem et al., 2021; Gaigher et al., 2022). Technology as a tool for communication was helpful for many and allowed contact with family and friends during the study period. However, some caregivers described how their loved ones with impaired brain health challenge and dementia in particular found such efforts disorientating. “Technology” as a new addition to care, compounded feelings of disconnectedness and thus additional difficulties. In addition, not everyone has access to technology (Gaigher et al., 2022) or it's connecting infrastructure.

## Limitations

This study has some limitations. There were no data on whether caregiver respondents provided in-home care for people with brain health difficulties or long term care facilities. There were insufficient numbers from each the four different English-speaking regions reported for an in-depth subgroup analysis by country, and the majority of respondents were from the US. There were a variety of lockdown policies and practices within countries and regions with internal variations over the course of the pandemic. While it is beyond the scope of this paper to include these variables in our analysis, we recognize that differential national/regional health systems and regional lockdowns policies and practices would be implicated in caregiver experiences. Nevertheless, this represents the largest, cross-country survey on the impact of the COVID-19 pandemic on caregivers of people with brain health challenges to date. It serves as an important resource for support agencies and to inform policy.

## Recommendations

The impact and implications of adverse events and global crises inevitably exacerbate difficulties of already at-risk populations. It is important to acknowledge and understand caregivers' distinctive vulnerabilities on an on-going basis. There is a need for policy recognition of the essential role of caregivers in combating isolation (Hindmarch et al., 2021). Support agencies should continue to provide virtual support, as also suggested by Boamah et al. (2024). There is a general move toward digital connectivity, that could lead to new opportunities in fostering brain health (Hachinski et al., 2021). Although the rapid transition to digital service structures in the social and health sector seemed essential during the pandemic, there may be challenges as Abrams and Szefler (2020) suggest, online-only services and -counseling offerings may be problematic, regardless of the presence of a pandemic. It is important to foster consistent social support and opportunities to maintain social connection are vital to supporting positive physical and emotional health and wellbeing (Jordan, 2023). The importance of social interaction was clear through its absence as personal social networks and opportunities for external engagement were limited during this time, and the recognized need for physical, psychological and social wellbeing intensified (Hachinski et al., 2021).

## Conclusion

The issues highlighted in this cross-national qualitative analysis were identified from responses provided by the caregivers themselves. Practical, social, psychological and emotional impacts affected their ability to offer care during the pandemic period. There was clear evidence that the disruption to health and social care services—institutions, day care and in-home services—impacted on usual care routines. The disruption produced practical and psycho-emotional challenges for both caregiver and care recipient. During the pandemic, caregivers' own contexts changed in so far as some lost employment, others worked from home, schools were closed in several jurisdictions and children and other family members remained at home. There was less social contact to enhance interpersonal engagement and at-home intergenerational care duties for multiple family members generated more complex caregiving responsibilities. Respondents also identified how changes in the care recipient—deterioration in their health and progressions of disease—reduced their ability to provide care. It is important to understand the implications of using standard communication technology with people with impaired health e.g., screen viewing, hearing and engaging within an impersonal medium may negatively impact people with sensory impairments and brain health challenges. There were national variations in restrictions associated with COVID-19 and frequency and duration of lockdowns in the countries represented in this respondent group. Nevertheless, many of the factors implicated as social determinants of health were negatively skewed among the caregivers surveyed during this prolonged period (2020–2021). The insights identified by the caregivers in this study should serve as valuable contributions to inform policy and practice guiding health and social care changes. This study underscores the importance of prioritizing the health and wellbeing of this indispensable informal caregiver workforce.

## Data availability statement

The data analyzed in this study is subject to the following licenses/restrictions. Restrictions apply to the availability of the CLIC data, contact the senior author (MG) for further information. Requests to access these datasets should be directed at: galvinmi@tcd.ie.

## Ethics statement

The studies involving humans were approved by University of Ulster Ethics Committee. The studies were conducted in accordance with the local legislation and institutional requirements. The participants provided their written informed consent to participate in this study.

## Author contributions

PK: Project administration, Analysis, Writing – original draft, Writing – review & editing. CR: Project administration, Data curation, Analysis, Writing – original draft. DH: Project administration, Data curation, Analysis, Writing – original draft. YC: Conceptualization, Data curation, Analysis, Writing – original draft. EG: Analysis, Writing – original draft. IL: Conceptualization, Analysis, Writing – original draft, Writing – review & editing. AW: Analysis, Writing – original draft. MG: Project administration, Methodology, Supervision, Conceptualization, Analysis, Writing – original draft, Writing – review & editing.
